# Adults With Special Educational Needs Participating in Interactive Learning Environments in Adult Education: Educational, Social, and Personal Improvements. A Case Study

**DOI:** 10.3389/fpsyg.2021.662867

**Published:** 2021-05-26

**Authors:** Javier Díez-Palomar, María del Socorro Ocampo Castillo, Ariadna Munté Pascual, Esther Oliver

**Affiliations:** ^1^Department of Linguistic and Literary Education and Teaching and Learning of Experimental Sciences and Mathematics, Universitat de Barcelona, Barcelona, Spain; ^2^Instituto de Mediación Pedagógica, Mexico City, Mexico; ^3^Social Work Training and Research Section, Universitat de Barcelona, Barcelona, Spain; ^4^Department of Sociology, Universitat de Barcelona, Barcelona, Spain

**Keywords:** adult education, interactive learning environments, dialogic learning, inclusion, well-being

## Abstract

Previous scientific contributions show that interactive learning environments have contributed to promoting learners' learning and development, as interaction and dialogue are key components of learning. When it comes to students with special needs, increasing evidence has demonstrated learning improvements through interaction and dialogue. However, most research focuses on children's education, and there is less evidence of how these learning environments can promote inclusion in adult learners with SEN. This article is addressed to analyse a case study of an interactive learning environment shared by adults with and without special needs. This case shows several improvements identified by adult learners with special needs participating in this study. Based on a documental analysis and a qualitative study, this study analyses a context of participatory and dialogic adult education. From the analysis undertaken, the main results highlight some improvements identified in the lives of these adult women and men with SEN, covering educational improvements, increased feeling of social inclusion, and enhanced well-being.

## Introduction

The World Health Organisation estimates that more than one billion people live with some form of disability (WHO, 2020), corresponding to ~15% of the world's population. It states that 3.8% of people aged 15 years and older have significant functioning difficulties and require assistance from various services. Furthermore, according to UNESCO, people with disabilities are more likely to be out of school or drop out of school before completing primary or secondary education (UNESCO UIL | UNESCO Institute for Lifelong Learning UIS, 2017).

According to UNESCO (2019), adults with disabilities are considered one of the most vulnerable groups in society. The limited possibilities to attend or complete school as children led to low literacy capacity as adults and overall educational achievement, which negatively influences their participation in further education following the Mathew Effect, which states that those with more education get more. Those with less education get little or nothing. Adults living with disabilities are increasingly being a target group for adult learning and teaching in different countries. However, they are still poorly visible and continue facing barriers in accessing adult learning and education.

To achieve the fourth goal of the Sustainable Development Goals (UN, 2015) (ensuring inclusive, equitable and quality education and promoting lifelong learning opportunities), it is necessary to investigate which educational actions serve this purpose, in which contexts they occur, and the role that adult education can have in it.

In this article, “adult education” is used in a sense given to it by the international scientific community at CONFINTEA V (UNESCO, 1997), as it can be read in the *Hamburg Declaration*. The impact of the fifth *Conférence Internationale sur l'Education des Adultes (CONFINTEA V)* held in this German city in 1997 in the definition of EU policies on adult education and lifelong learning is relevant to mention. In the Hamburg event (1997) many debates were held about the role of adult education in a changing environment, being adult learning understood as an integral part of lifelong learning. Learners were conceptualised as subjects (not objects) of their learning processes and adult education was connected to community learning and to dialogue between cultures. Adult education was related to social and economic development struggles, to justice and equality, being a potential way for individual empowerment and social transformation (Oliver, 2010). CONFINTEA VI (2009) continued being a relevant platform to further dialogue about formal and non-formal adult learning policies at the international level, establishing ambitious goals and urging to real actions towards advancing to favour that adults enjoy their human right of lifelong learning. In the next future, CONFINTEA VII (2022) will continue this line contributing to the analysis of efficient learning and adult education policies from the lifelong learning perspectives, taking into account the Sustainable Development Goals from the United Nations (UNESCO). Thus, this understanding of adult education is also related to the idea of democracy, social justice and solidarity that some communities are promoting to enhance the learning opportunities for all students (Vanegas et al., 2019).

In that sense, adult education encompasses formal, non-formal and the whole range of informal and occasional learning in multicultural societies. This concept includes diverse learning spaces, among others: home, school, community and workplace.

Key historical and political milestones influence the development of the adult education in Europe.

The *White Paper in Education* (European Commission, 1995) represented a relevant moment in the understanding of the advancement of Adult Education policies in the European Union. It signified the promotion of education and training in Europe in a context of technological and economic change, proposing objectives to guarantee a high-quality education for all. Specific EC action programs, such as the Socrates programme with a section of adult education, were an important milestone in this context, followed by the Grundtvig action, focused on adult education and other educational pathways to promote lifelong learning with a European dimension.

In 2001, the European Ministers of Education defined the main goals to be achieved, including improving the quality and effectiveness of educational and training systems. At that moment, it was already recognised that people with more difficulties to be engaged in lifelong learning processes had a greater risk of suffering social exclusion (Council of the European Union, 2001). This implied efforts to promote social inclusion in AE, to overcome barriers and favour more significant access to different educational and training systems for all. The case analysed in this article is also addressed to show how several of these barriers can be overcome through a concrete interactive learning environment in the case of the adult learners participating in the study.

Similarly, in line to favour lifelong learning strategies across Europe, the Memorandum on Lifelong Learning (Commission of the European Communities, 2000) launched a consultation process across Europe to identify strategies and ways to foster lifelong learning opportunities for all. Lifelong learning was considered an umbrella for a wide diversity of learning processes, from pre-school to post-retirement, including informal and non-formal learning. From this process of consultation, the establishment of a European area of Lifelong Learning was proposed. It was thought to create a common frame in Europe to facilitate mobility and more coherent use of the existing resources towards lifelong learning, promoting the centrality of the learner within the learning process, equal opportunities and the quality and relevance of learning opportunities (Commission of the European Communities, 2001). Relevant stress for analysing learning needs more precisely and to respond to the needs of diverse social groups was identified. In 2006, for example, the EC Communication *It is never too late to learn* (Commission of the European Communities, 2006) encouraged the Member States to increase and consolidate lifelong learning opportunities for adults and make them accessible. This article responds to the need to provide scientific evidence on the improvements of a concrete interactive learning environment in specific learning and personal trajectories of adults with special needs. Since the Council Resolution on a renewed European agenda for adult learning (Council of the European Union, 2011), relevant emphasis was given to promote the acquisition of work skills, active citizenship and personal development and fulfilment, favouring flexible learning environments and mechanisms to assist adult learners.

Consequently, today Adult Education is intrinsically linked to lifelong learning, affects the actors involved and envisages the extension of multiple educational networks encompassing all possible institutions. Adult education understood as a common good is achieved in a society when there are accessibility, availability, affordability and social commitment to its functioning (Boyadjieva and Ilieva-Trichkova, 2018).

According to previous research (Desjardins, 2019; Hamdan et al., 2019) adult education has positive effects on a wide range of aspects, such as adult empowerment, social inclusion, social networking, motivation for learning, work-related aspects, including improved job and career prospects, performance and earnings, job satisfaction and commitment to work and innovative skills, as well as other parts of everyday life (Moni et al., 2011; Ryan and Griffiths, 2015; Magro, 2019).

Adult education can also have an impact on adults with special educational needs. By “adults with special educational needs” we mean people who have long-term physical, mental, intellectual, or sensory impairments which, in interaction with various barriers, may prevent their full and effective participation in society on an equal basis with others (UN, 2006). Recent research in education suggests that learning environments based on inclusive interactions help promote learning and development of students with Special Educational Needs (SEN).

In the case of children with special educational needs, previous research suggests that their participation in educational activities developed in inclusive, interactive environments has clear benefits on learning (Duque et al., 2020). However, this result has not yet been discussed in the case of adults.

According to the findings of Moni et al. (2011) with adults with SEN in community-based adult education contexts, community organisations contribute to the literacy processes of participants with SEN in these programmes. This study points out that, for many years, functional skills training (such as cooking and manual jobs) has dominated community-based programmes for people with SEN and there has been limited recognition of the role that literacy can play in improving the quality of life of learners with SEN through lifelong learning (p. 474). There is currently no research investigating the degree of literacy needed by adults with SEN in a variety of contexts in adulthood. Depending on the adults' needs, literacy needs can vary widely from employment, family, daily living challenges, leisure and recreation, even to the degree of literacy needed in specific areas such as computers/internet and the broad area of health issues. In any case, it is a basic instrumental knowledge necessary in diverse contexts; therefore it is relevant to identify venues to enhance its learning.

The development of social competences is an integral part of education of this collective. According to de Morais and Rapsová (2019), several specific criteria have to be considered when working with people with special educational needs. Some of them are: (1) To perceive the education of older people as a lifelong process, (2) to take into account the possibilities of education in the system, (3) to recognise the needs and interests of individuals, (4) to enable education without discrimination, (5) to improve the quality of life through education and occupations, and (6) to make use of their life experience for themselves and society as an asset (de Morais and Rapsová, 2019).

In this sense, training focused on social aspects can be beneficial because competences to manage a wide range of social situations provide specific protection in cases of stress, tensions and conflicts. A reasonable level of social competences significantly determines the ability to cope with everyday stress, create excellent and non-conflictual interpersonal relationships, and find more efficient ways of resolving conflicts and misunderstandings. Socially competent people play an active role in their lives, can express their needs and achieve their personal goals (Wilkinson and Canter, 2005; Praško et al., 2007).

Some studies focus on analysing the participation of adults with SEN in training and lifelong learning activities from a labour economic perspective (Myklebust and Båtevik, 2014; Båtevik, 2019) and highlight the value of receiving formal education for the acquisition of future employment opportunities. However, these studies do not delve into the educational characteristics of such learning opportunities for this specific group.

Other research highlights the importance of collaborative work between caregivers of people with learning difficulties and educators in charge of training programmes as this raises awareness of the value of education for these adults and facilitates the establishment of learning opportunities in the everyday lives of people with learning difficulties (Wilson and Hunter, 2010; Brown, 2020).

It is known that interaction and dialogue are critical components of learning (Flecha, 2000; Aubert et al., 2009; Racionero, 2017). Following the sociocultural theory of learning initiated by Vygotsky, learning and cognitive development are explained as cultural processes that occur in interaction with others (Vygotsky, 1978; Rogoff, 1993). Specifically, Vygotsky develops how the human learning is understood as presupposing a specific social nature and a process by which children grow into an intellectual life of those around them (Vygotsky, 1978: 78). Similarly, Bruner (1996) also highlights that learning is an interactive process in which people learn from each other and Wells (1999) argues about the way human beings built their knowledge about the world through a common action and about the way this knowledge is later used in their collective action.

Subsequently, a dialogic turn in educational psychology (Racionero and Padrós, 2010) explained that interactive and dialogical learning environments improve students' learning opportunities and outcomes. The project *INCLUD-ED: Strategies for Inclusion and Social Cohesion in Europe from Education* identified a set of Successful Educational Actions (SEAs) (Flecha, 2015) that have been shown to contribute to improved learning outcomes and social cohesion (Soler-Gallart and Rodrigues de Mello, 2020). These SEAs have been shown to increase learning efficiency, i.e., instrumental tools needed to live included in today's society (basic and transversal skills), and generate equity. Subsequent research has reinforced this evidence, showing that organising teaching based on interaction and dialogue simultaneously improves performance and coexistence among the student group (García-Carrión et al., 2016). Interactive Groups and Dialogical Gatherings are two of the SEAs that allow this type of teaching organisation to be carried out so that high levels of learning are achieved in safe and supportive spaces that promote friendly relationships and better coexistence. Interactive groups -IGs- (Valls and Kyriakides, 2013) are a way to organise the classroom in which the students are split in groups, with a volunteer facilitating that all participants in the group interact with each other when solving the task. IGs draw on to the principles set up by the “Dialogic Learning” theory (Flecha, 2000), that is: participants engage in an egalitarian dialogue in which they exchange statements (arguments, reasons, facts, etc.) drawing on validity claims, rather than on their “power” position within the group. Dialogical Gatherings work on the basis of dialogic reading: participants read universal readings, and then they share their reading in a gathering, where everyone can contribute reading aloud the fragment they want to share. Then all participants in the gathering can comment or discuss on the fragment, reaching a distributed (Hutchins, 2000) understanding of it throughout the dialogue (Bakhtin, 2010, Flecha, 2000). Dialogic Gatherings include different types, such as Dialogic Literary Gatherings (participants use universal readings), Dialogic Music Gatherings (participants use universal plays), Dialogic Mathematics Readings (participants read mathematics masterpieces), Dialogic Arts Gatherings (participants share their comments on universal paintings or sculpture), etc.

These contributions also apply to students with disabilities, as they benefit from interactive learning contexts to progress to higher levels of learning and higher stages of development. Duque et al. (2020) state that interaction and dialogue positively impact students with SEN. According to the results they present, participating in activities such as interactive groups or dialogical discussions with the rest of the students, makes students with SEN improve their learning and social integration skills with the rest of the group. Interacting with peers with higher academic competence levels under the same curriculum allows students with special needs to make more significant learning progress in mainstream schools. Each person, regardless of their condition, can contribute from their cultural intelligence to the learning process. Previous research suggests that placing students with SEN in the mainstream classroom, together with the rest of their peers, and promoting interactions based on egalitarian dialogue (Flecha, 2000), has benefits both on the learning of students with SEN and the rest of the students (Fernandez-Villardon et al., 2020). Inclusion fosters the acquisition of academic skills (Dessemontet et al., 2012), improves educational outcomes (Nahmias et al., 2014) and intellectual engagement (Mortier et al., 2009) of students with SEN. It also has positive impacts on social development, as interacting with the rest of the student body leads these students with SEN to improve their social skills and the acceptance they receive from other students (Meadan and Monda-Amaya, 2008; Draper et al., 2019; García-Carrión et al., 2019).

Research also includes the analysis of how interactive learning environments are developed in special schools to create better learning opportunities for children with SEN. The results put forward by the authors suggest that rethinking the learning context by introducing interaction-based instructional models' benefits children with disabilities and provides high-quality learning and safe and supportive relationships for these students, thus promoting their educational and social inclusion (García-Carrión et al., 2019). However, such research is usually focused on children, so there is a gap in education for adults with SEN. This paper discusses the improvements of the case study's dialogical education context on the adult with SEN who have participated in this study. The aim is to identify these concrete adult learners' improvements in this interactive environment in terms of instrumental learning, social integration and personal development.

## Materials and Methods

This study is based on the communicative methodology (Gómez et al., 2011), which has been used in previous research that has achieved social impact with vulnerable populations (Puigvert et al., 2012), including adults and people with special educational needs (Duque et al., 2020). In the communicative methodology, an inter-subjective dialogue is established between the people who participate in the research and the researchers, from the design of the study to the interpretation of the results (Gómez, 2019). In this dialogue, international scientific evidence is contrasted with the participants' everyday experiences, which allows for the construction of new scientific knowledge that is useful for transforming the analysed realities (Flecha, 2014; Flecha and Soler, 2014). This methodology contributes to providing solutions to the problems faced by citizens in different social areas, including education and social inclusion (Soler and Gómez, 2020; Torras-Gómez et al., 2019).

### Description of the Case

Following the postulates of the communicative methodology, this research has been developed as a case study (Flyvbjerg, 2006; Yin, 2011). This case study aims to analyse how this type of learning environment promotes inclusion, educational improvements and enhance well-being in adult learners with SEN participating in this study. In social sciences, case studies are one of the principal means in which research is carried out. For this research, the case study allowed an in-depth exploration from multiple perspectives of the complexity and uniqueness of a particular *real life* educative context (Simons, 2009). The case study is about La Verneda Sant-Marti school, a school for adults located in Barcelona, Spain (Sánchez Aroca, 1999). This school is a Learning Community (Soler-Gallart and Rodrigues de Mello, 2020), the first of its kind, and implements Successful Educational Actions (Flecha, 2015). The school was created in 1978 in response to the demands of neighbourhood residents. Since then, it has continuously taught people to read and write, helping adults obtain academic qualifications that facilitate their insertion into the labour market or promoted their access to university, and their fully participation in civil society. The school is an international reference for its trajectory and contributions to the democratic movement in education (Sánchez Aroca, 1999; Aubert et al., 2016) and is, precisely, for that reasons that was selected as case study in this research. This antecedent allowed the research team to explore this case, paying particular attention to the dynamics and characteristics of the school that are linked to the educational and dialogic participation of adults with special educational needs. The school is the result of the empowerment of the neighbourhood; it was founded by citizens encouraged to learn and access education, and thanks to volunteering, they manage to organise what today is the Learning Community of the La Verneda Adult School- Sant Martí.

The didactical and methodological organisation approach followed by the school is called “dialogic learning” (Flecha, 2000). As Flecha (2000) explains in his book, “Dialogic learning” is based on seven principles: egalitarian dialogue, cultural intelligence, transformation, instrumental learning, meaning creation, solidarity and equality of differences. Adult learners engage in egalitarian dialogue, exchanging their understanding (based on their previous personal, professional, cultural experience) around the topics discussed/learned within the lesson. Teachers empower adults to engage in this particular way to interact with each other, encouraging adult learners who find more difficult (or challenging) to participate, share their points of view, and thus generate more opportunities for interaction through the exchange of dialogue. All participants in the lesson can contribute to the learning process since all of them (all of us) have “cultural intelligence.” This “cultural intelligence” is mediated by personal experiences, as well as knowledge acquired within the workplace, for belonging to a particular cultural group, … Learning becomes a solidarity process in which adults share their own sources of understanding, creating avenues for enriching their collective understanding of the topics discussed/learnt within the lesson. Dialogue becomes the way to share all these “meanings.”

This school counts with seven fulltime workers and 120 volunteers who are in charge of facilitating the school's courses and training activities (Aubert et al., 2016). The school is organised by two associations that are an integral part of the school's educational project: Ágora and Heura (the latter is specifically for women) (op. cit.). In this way, the school can be classified as a non-governmental organisation. The number of students with SEN are about 30. They include both people with physical disabilities as well as people with cognitive NEE. One of the latter is also a member of the school board, and he participates fully in the decision-making process regarding school issues. Table 1 summarises the population of adults with NEE participating in this school.

**Table 1:** Distribution of the adults with NEE participating in the adult school, by course.

**Course**	**Number of participants**
Beginners (neo-literacy and numeracy courses; people who is learning who to read, write, and perform basic arithmetic calculations)	7
Secondary education	4
Access to the university (training to apply to the exam that the Spanish universities facilitate for people older than 25 years old without previous academic degree)	2
Catalan as a second language and Spanish as a second language (for migrants)	6
Dialogic Literary Gatherings	9
Sing language course	1
Other courses	1

### Data Collection Techniques and Participants

The information collection techniques used to conduct this qualitative case study consisted of documentary review (Stake, 2013) of files referring to the school's organisation that were requested from the administration and others obtained from the information, that the same school publishes on its website. Likewise, scientific articles published about this educational centre were explored to understand the educational context of the school, the forms of democratic organisation under which it is managed and the pedagogical principles that govern its educational activities linked to the participation of people with special education needs.

Another data collection technique used was semi-structured interviews to establish an open and in-depth dialogue between researchers and research participants. From the communicative approach (Flecha, 2014; Flecha and Soler, 2014), the semi-structured interview aims to establish a dialogue between the person doing the research and the person participating in the study, to reflect on and interpret the phenomenon or object of study. These interviews were carried out from an orientation scrip. According to Denzin and Lincoln (2011), in a case study each case has “value” in their own. It is not expected to generalise, but to provide an analysis of the research topic based on selecting participants that are “significant” because of their experiences, expertise or personal knowledge about the research topic. The people participating in the field work were selected responding to the following profiles, and according to their availability and acceptance to participate in the study: adult people with SEN, school workers (volunteer teachers), social workers (specialised personnel to work with people with SEN), occupational therapists from a mental health centre that collaborates with the adult school. The diversity of profiles allows a triangulation of data to enhance the validity of the results. The profiles of the interviewees are detailed in the Table 2.

**Table 2:** Participants.

**Pseudonym**	**Profile**	**Duration**
Manolo	Student with schizophrenia	26 min 20 s
Carolina	Student with mild intellectual disability	22 min 11 s
Andrea	Teacher	35 min 44 s
Isabel	Social worker	15 min 49 s
Cintia	Teacher	27 min 27 s

The semi-structured interviews were conducted through telephone, and the audio was recorded. The first contact with the participants with SEN was made by a teacher at the adult school, who has a close relationship with them. This methodological decision was made to ensure an environment of trust allowing the interviewees to feel safe participating in this study. One member of the research team, who already knew some of the adults participating in the study, conducted the interviews. One of the participants with SEN refused to be interviewed by other that the teacher with whom he had confidence. Thus, we asked the teacher to conduct the semi-structured interview. We explained the objective of the study to this teacher, as well as the script to carry out the semi-structured interview. All participants were informed before the interview about the aims of the study and gave their oral consent to participate. All personal details have been securely stored, and no real names are used, for confidentiality reasons, in order to protect the identity of the participants. All names used in this article are pseudonyms. The study was fully approved by the Ethics Board of the Community of Researchers on Excellence for All (CREA).

### Data Analysis

The communicative methodology (Gómez, 2019) has two dimensions, the exclusionary and the transformative, which reflects, respectively, the components that prevent or help social transformation. In our case, the analysis of the data from these two dimensions allows us to identify, on the one hand, the transformative elements that explain or intervene in the impact that interactive learning environments have on adults with SEN and the features that hinder this impact (Pulido et al., 2014). For the analysis of the data, categories of analysis were established based on the study objectives: (1) instrumental learning, which covers educational improvements and contributes to progress in their academic training, (2) social integration, which allows the learners to participate actively and promotes the development of communicative and practical skills, (3) personal development, which is linked to attitudes of empowerment, confidence and improvement of individual skills. These three categories were analysed in terms of the two dimensions of analysis mentioned above: exclusionary and transformative.

## Results

### An Educational Centre Open to the Participation of Adults With SEN Through Successful Educational Actions

The Verneda Sant Martí Adult School is a democratic and plural project where decisions are made by all the people involved in the community through participation, dialogue and consensus. In this school, to participate is to intervene, take part, contribute, listen, be heard and act in all areas and spaces of the school: in the classroom, in committees, in preparing the agendas for meetings, etc. Participation is understood as an attitude that includes all people, all spaces and all processes from the beginning to the end. Dialogue and consensus are the basis for the organisation through deliberative democracy (Habermas, 1992). This dialogue and consensus include social and cultural plurality to build agreements that ensure that decisions and actions can be valid beyond a closest environment.

As mentioned, this school operates according to the dialogic learning principles (Flecha, 2000) and is characterised by the following aspects (Aubert et al., 2016):

1. Non-academic adults participate in all decision-making processes; therefore, all activities reflect their interests and needs, increasing their educational level and skills.
2. The school is open to the community and has engaged many diverse people as volunteers who contribute to a broad and high-quality education.
3. The democratic organisation of libertarian origins influences the School walls: a neighbourhood movement to improve the quality of life and the transformation of schools into Learning Communities.

According to previous studies (Serrano, 2015; Aubert et al., 2016; León-Jiménez, 2020), the key to its success is an effective democratic organisation and functioning, developing a wide variety of activities and an accessible timetable. Adult participants, together with teachers and volunteers, decide and organise the activities to be carried out in the school according to their needs and interests.

This school has always had participants with disabilities and other special needs who have participated in the school's activities on a regular basis. Due to the school's interactive, democratic, and participatory nature, students with SEN are not segregated, neither inside nor outside the group, and teaching is based on a high expectations' basis. Previous research (Molina, 2015) highlighted that Learning Communities promote the inclusion of people with SEN through their inclusive and equal participation in activities shared with the rest of the students, in heterogeneous groups. Interactive groups and Dialogic Gatherings are examples of those kinds of groups.

The interviews carried out show that the school opens its doors to the participation of diverse students, receiving adult students with special educational needs from other entities, health organisations specialised in working with people facing some kind of disability and neighbours from the same neighbourhood who are interested in participating in the school.

Cintia: In fact, we have cases because they come to us from organisations, for example, people like Carolina, or Mohammed, and others who participate in secondary education graduate courses (…) and others who are in initial levels, several in the afternoon neo-literacy courses (…) we get people with mental health problems, but also with a degree of disability. They start by participating in a discussion group, and from there the moderator suggests to them to study something or participate as a volunteer in the school.

As a Learning Community, the school promotes interactive learning environments by implementing Successful Educational Actions in workshops, courses and learning spaces. The didactic and methodological organisation in interactive learning means that all participants have the same opportunities to contribute to and participate in the learning experience. They engage in an “egalitarian dialogue,” in the sense that everyone can share their own statements drawing on “validity claims,” rather than other sources of argumentation (such as “power claims,” in *habermasian* terms). Participants in a Learning Community are very diverse (heterogenic), meaning that they engage in the interactive learning experience drawing on different types of “understanding;” since all of them are endorsed by “validity claims,” participants have the opportunity to enrich their learning experience incorporating different ways to achieve this “understanding” about the topic discussed in the lesson. In this way, people with SEN participate equally in the construction of learning.

The school carries out SEAs such as Dialogic Gatherings or Interactive Groups. In the Interactive Groups, students with special educational needs interact on an equal basis with other people and based on mutual help and solidarity, learning is generated. Solidarity is understood as a relevant component of adult education that has a transformative aim. Actually, it is one of the seven principles defining the “dialogic learning” approach (Flecha, 2000). In the following quotes, one of the volunteers explains these interactions and highlights the fact that the integration of adults with SEN with the rest of the group, without exclusion or segregation, is based on the fact that the highest expectations are placed on the learning of all people:

Cintia: Actually, it's like that of any other person in the class. Because we organise ourselves in Interactive Groups, so when I teach maths, we try to make the groups diverse, heterogeneous, and I think of Mohammed, who is quite good at maths. Like anyone else, he does the activities and helps other people. And the other way round too. Everyone helps with what they find more straightforward and with what they find more difficult, they help them.

Cintia: Also, the fact of organising the class in Interactive Groups, in this way encourages them to help each other, and maybe one person with special educational needs can explain the meaning of a word in Spanish, and another person can help them with something else. They are like anyone else in the group, and they learn just like the others. And so, everybody is getting to the same level of learning (…) And the fact that they are the same as everybody else. If there may be a need to have a space to extend the learning time, but not separated from the group, but with the maximum expectations.

Adults with NEE also participate in Dialogic Gatherings, such as the Dialogic Mathematics Gatherings (Díez-Palomar, 2020). They share and enjoy their readings on masterpieces about singular mathematicians with their peer in the group. Carolina, for instance, highlights her participation in different types of dialogic gatherings, of which she is proud to be part:

Interviewer: In what type of activities do you participate?

Carolina: Dialogic Mathematics Gatherings. In summer: photonics (Dialogic Science Gatherings). Dialogic Women Gatherings. Cultural Gatherings. I also participated in a seminar on Astronomy. We came to “La Pau” [this is a neighbourhood next to the La Verneda – Adult School]. In class, we also participated in this about women, the Cultural Gathering.

The inclusive configuration of the SEAs also favours that people with SEN, who sometimes face more significant communicative challenges when establishing relationships with diverse people, find an opportunity to develop their social skills. This effect also applies to volunteers who, by interacting with people with different abilities, have the chance to learn from them and overcome prejudices or stereotypes about disabilities:

Andrea: I see that the IGs were relating to other people who were different. Because those people only left the residence to go to school, and you take them out of their comfort zone, and you force them to change the kind of relationship they are used to. Maybe they are people who in their day-to-day life would not relate to this type of people, but the IG forces you to connect to them, and it also helps you to break with prejudices you had before. For example, the infantilisation, that this person does not believe that s/he is able to do that, but in the end he/she does it, and by different ways, they can reach the same goal, and the prejudices are broken.

### Improving Learning and Advancing Academic Training

According to the people interviewed, both the participants with SEN and the volunteers who work at the school, the participation of these adults with SEN in the same activities as the rest of the people who go to school results in an improvement in their instrumental learning. These improvements are manifested as discipline-specific learning. In the following quote, one of the participants interviewed mentions the learning she has acquired from participating in various Dialogic Gatherings and courses in this school.

Carolina: You learn a lot, for example, in mathematics, you learn about mathematics, which is curious; about women, the problem of gender violence, about photonics you learn about lasers, physics, chemistry, and many cultures … A lot of things. Things you've never heard of before. It's good for your memory. I love to participate.

The improvement of learning is also evident in the achievement of certifications or accreditations that allow them to continue with higher-level academic training or that enable them to enter the labour market.

Cintia: And also, in terms of employment, because, for example, technology training is essential. Or like Manolo, who has passed the entrance exam and is now studying political science [at the university].

The centre's volunteers also provide examples of improved learning in terms of acquiring basic reading, writing and technology skills of students with disabilities who have participated in the school. In the quotes presented, the volunteers highlight the help and solidarity provided by their colleagues at the centre as an essential factor in the achievement of learning:

Cintia: a man with deafness was participating in an online course, and despite being deaf, by the fact that he helped his colleagues, he was able to have the certificate of the course, which will allow him to have the necessary papers, etc.

Isabel: For example, people who have come here, we have worked with them, they have taken entrance exams [to the university] for people over 25s years old, they have been able to pass their university entrance exams, and they have ended up as volunteer trainers in ICTs too.

Andrea: What I saw most was with a person with a physical disability, who had low vision. I saw quite a big impact. At the time of adapting the material and also with his classmates, with his informed consent, they saw that he made great progress in terms of learning. And even above all the support of his classmates, as the central axis, that they read to him the things that he didn't understand, and when he finished the course, his level of Spanish went up a lot, and it came out that you could hold a conversation with him. He did very well.

On the other hand, the interaction with more people for more extended periods have allowed people with SEN participating in the centre to develop other cognitive, communicative and social skills. In the following quote, one of the volunteers at the centre highlights these improvements:

Isabel: On a cognitive level, there is also an improvement or training of processing skills, tolerance, planning, and they also work on their commitment to themselves and the group. So, there are a series of integral improvements.

This acquisition of social competencies such as self-regulation and coexistence is also highlighted by one of the students interviewed.

Manolo: When you have 20 or 30 older people, the demands are more challenging because you expose yourself directly to the public, and what you try to do is prevent the exposure to the public. That exposure went very well. I didn't have any problems beyond something that was inside me. I had to go outside, go to the bathroom, cold water, cold breaths, and I came back, and I was normal, and little by little, you overcome these things, you learn to control them.

### Social Inclusion Through Dialogue and Democratic Participation in Interactive Learning Environments

The participation of adults with SEN also extends to their involvement in the educational life of the centre. The following quote reveals how some of the people with SEN are participants in the academic spaces opened by the centre and become volunteers in another space, being them who contribute to other people's learning:

Cintia: Manolo started with the mental health centre. They suggested he come to the school, he did the university entrance exam and began to collaborate in the course about how to use a smartphone, he volunteered. And we had another case of a boy who came to collaborate teaching sign language classes. And Paco, who has been in the school for many years in the neo-readers group, also in computer groups. Last year, he also collaborated teaching other people at the initial level of teaching computers. He is also on the school board.

Also, for the participants, the school represents a place that differs from other contexts in the sense that it allows participants to be involved in the creation of the educational content they receive and gives a sense of warmth:

Manolo: a school like this contributes a lot. They do, but there are other adult schools, which I don't know if they belong to the *Generalitat* [Catalan Government] or what, and they have nothing to do with it. Adult schools that are not like this school, everything is very mechanised. The management is much colder. Not here, here it is the community itself that creates the content.

Equal treatment is also identified as an essential element in the life of the centre and is present in the interactions established in educational spaces:

Andrea: When they are people with functional diversity, we always try to include a supporting person … then a very individualised support also takes place, treating them as equals and making them participate in the whole process because they can decide on everything, to continue, not to continue, whether to change the course or not … For example … to literacy, which is a course of initial level … if that person talking to her says: you are ready to move to neo-literacy … talking to her … and not deciding for her, that is also very important.

In one of the students' testimony, the equal treatment he has experienced when participating in the school with other adults is highlighted. This equal treatment does not deny the differences between the different learners, but neither does it reject them. The interactions that are established between students with SEN allow them to feel part of the group and to relate to each other on an equal basis based on their differences:

Manolo: It was quite essential. If you behave in a regular way, people treat you in the usual way. It was something Albert knew. When I started teaching there, I would say it in class, and you could see how people's reaction was surprising, but not rejection, and with the time that becomes normal and they don't reject you, but they don't adore you either, you're just one of them.

The school's volunteers also recognise these egalitarian dynamics based on the inclusion of all the people participating in the school. In her testimony, one of the school's volunteers states that it is difficult to identify students with SEN because they are all participating in the same space and under the same educational conditions. Such an environment is conducive to overcoming stigmas and social stereotypes.

Andrea: At the time, I didn't know exactly which ones I had, and they were starting to do the tests. And another one of functional and mental disability. He participates in the computer and computer classes, and as a volunteer, he will help us with the computer in class and when we need help with laptops.

Isabel: on a social level, it allows them real contact with people without having the stigma component in between.

The democratic and egalitarian management of the school also stands out as a critical element for the inclusion of people with SEN. The analysis of the information collected allowed us to identify that the democratic processes under which the school is managed are a critical factor in the inclusion of adults with SEN. This type of participation generates motivation and interest, given that the educational offer provided responds to the needs of the people involved, and it is decided democratically. It also favours the development of personal skills such as decision-making, organisation and collaborative work. Both volunteers and participants highlight these aspects:

Cintia: Democratic management of the whole school, the same people involved, including people with disabilities, are the ones who make all the decisions (board, assembly, school council…) or when we do projects they are also there, and this ensures that the things they do take into account their needs. For example, Paco (who makes sure that things work).

Participating in organisational aspects at school enables people with SEN to develop skills and values useful for their functionality in every day and working life. In the following quote, one of the students with SEN interviewed explains how his participation in school helped him in acquiring new social competences in addition to the academic dimension:

Manolo: Yes, because on the one hand it prepared me academically for what I wanted, which was to go to university, and on the other hand, as a collaborator, it favoured me in many personal aspects, it empowered me much more than just passing the grade, more profound things, in the day to day, being responsible for 20 people, who are interested in what you are telling them, and in such an altruistic way as in that school, is very enriching in all aspects.

Another important finding is that the school offers its programmes free of charge, as it operates through the voluntary and supportive participation of many people. This aspect is highlighted as relevant for the inclusion of adults with disabilities. In the following quotation, the interviewee alludes to this and states the importance of the fact that the school accepts any type of student regardless of their disability:

Isabel: First of all, to participate based on the criteria of universal access to education. Then the variable of the cost, free of charge, allows them to carry out training. Then working horizontally, where only the person is considered and not his/her dysfunctionality … just like the other participants, the accompaniment provided from the beginning, from health and educational resources. These are variables that can contribute to this (…)

Interviews with students with SEN also highlight that the solidarity-based and cost-free organisation of the centre facilitates participation in these learning spaces:

Manolo: In the entrance exam because I wanted to go to university.

Isabel: [talking about Manolo] He had no income. They did it for free. (…) he was admitted to a psychiatric unit, and he was in the forum. In terms of work abilities, he saw that it would be positive for him to collaborate in something with them, for his benefit, such as exposure to the public, habits, responsibilities. That's why. All derived from occupational therapy at the forum.

### Improvements in Personal Development: Bonding and Empowerment

The data analysis also reveals that the participation of people with SEN in interactive environments promotes bonding with others and contributes to their emotional well-being. These relationships transcend the school space to become part of their everyday social life. In the following quote, one of the participants interviewed gives an account of the friendships she has been able to establish as a result of her involvement in the school and how these are maintained outside the school space and form part of her support and trust networks:

Carolina: I have some of my classmates, with Nadia, with the teacher, with Irma, with my classmates, I talk about them by WhatsApp, and with you (maths discussions), through WhatsApp groups … now because there is covid-19, we congratulate each other at the end of the year by video call, I talk to my classmates (…) Or like Ruth, we did maths, and after that, we went to class. You can count on her; if you have a problem you can count on her, just like Ruth (from Dialogic Science Gatherings). Ruth has also helped me.

For their part, the volunteer participating in the research report about the improvements on the relationships of solidarity, in the terms of mutual support as is mentioned by the research participants, that are built through participation in these interactive learning environments:

Cintia: Relationship between colleagues. There is always a lot of solidarity between them. I think of the GES [Secondary Education], which is the group where I have been most of the time. And many times groups are created afterwards to study together, for example. In the GES group, where Carolina was, a participant who had passed the exam, the following year, they created a Catalan conversation group, and she helped them to study Catalan. They help each other beyond the classroom.

Finally, another aspect in the category of personal development identified in the research findings is the empowerment generated in adults with SEN as a result of their participation in these interactive learning environments.

Manolo: Yes. But it is something more general, and it is a concept that I call empowerment. It's not a specific thing that this lady taught me … it's a general thing. Why aren't you capable … when I came out of class, and you finished the lesson, you felt a sense of security, of power, because if you are capable of doing a class, what are you not going to be capable of? Another thing is if you want to be an astronaut … but for a normal life, that's useful. But then I also demand a lot of myself, etc. but that would be my internal things.

Cintia: Then, also at the level of empowerment, Paco has gone from not knowing how to read or write, to learning and being a member of the board, and he feels responsible for the school. For example, he makes sure that in *Omnia* [the computer classroom] there is always a newsletter of the activities, or that everything is up to date, and he is still very attentive and calls to know what is going on in the school (if 1 day he cannot come). For example, we know that the library will leave 1 day, and he is unequivocal “we have to get the space.”

These results provide evidence, based on the analysis of research participants' quotes, on the improvements in the learning processes, the feeling of social inclusion, and the well-being of the adults with SEN participating in this school. In the following section, these main findings are connected to previous scientific contributions to highlight elements from this school organised as an interactive learning environment relevant to improve adult with SEN well-being learning processes and their social environments.

## Discussion

Indeed, the results of this research show that, as with children (García-Carrión et al., 2016; Duque et al., 2020), learning environments based on interaction and dialogue are shown to be effective in achieving learning and progress in the educational trajectory also in the case of adults with SEN.

The people with SEN interviewed, who had not achieved literacy acquisition before, achieve literacy and continue to participate in more school-promoted programmes. Through interactive learning environments, these literacy processes are in line with research highlighting the value of literacy in achieving independence and well-being for adults with learning difficulties through collaborative work between schools and people or organisations that support this population (Wilson and Hunter, 2010).

Previous studies (Samuel et al., 2008) suggest that adults with SEN exposed to intensive interaction settings experiment a positive effect regarding their social abilities. They improve their relationships with other people, being more open to talking to others, engage in social situations, … Our data suggest that “cognition” can also experiment “improvements” as a result of social interactions in which adults with SEN engage when attending the courses/activities in the adult school. The participation of adults with SEN in interactive learning environments favours their progress in learning, as learning is offered from a transformative perspective with high expectations for all learners' learning. Some of the adult learners with SEN attending this school passed to higher education institutions successfully. This is the case of Manolo, the man that Cintia declares that was able to overcome his cognitive disability and passed the entrance exam to study political science at the university. Manolo felt empowered by the relationship with his peers and the teachers/volunteers in the adult school. The positive interactions (based in not segregating him and recognising his abilities) led Manolo to believe in himself and think that he could take the university entrance exam to study political science. This positive endorsement made that studying at the university (something that was not among his expectations before) become a reality. This result is in line with previous scientific research highlighting the importance of empowering adults with disabilities or learning difficulties to continue learning throughout their lives (Moriña, 2017; Lawson and Parker, 2019; Buchanan and Warwick, 2020).

Interactions with peers and with various members of the community enable personal development in terms of establishing relationships and the implementation of communication and prosocial skills by people with SEN (Villardón-Gallego et al., 2018; Magro, 2019). For the people who have participated in this study, the development of this type of social competences has positively improved their ability to cope with daily stress, create good and non-conflictive interpersonal relationships, and find more efficient ways of resolving conflicts and misunderstandings. Socially competent people play an active role in their lives, can express their needs and achieve their personal goals (Praško et al., 2007, Wilkinson and Canter, 2005). On the other hand, in the case explored, the interactive learning environments promoted through SEAs make it possible that, from these interactions, other people attending the adult school overcome stereotypes or prejudices about people with SEN, as reported by Andrea in her interview. Andrea explains how adults with SEN participate in an egalitarian basis within the interactive groups and the dialogic gatherings. They are exposed to situations that take them out of their comfort zone, thus being “forced” to change the type of relationship they use to establish with people without SEN. Our data suggests that this procedure can create avenues for adults with SEN to improve their social skills.

The results obtained are also consistent with what other research claims about the improvements in the participation of people with SEN in learning environments such as the school where we conducted this study; Ryan and Griffiths (2015), for example, found that in social inclusion settings that promote people with SEN interacting with others who do not have SEN, those people with SEN also participate in decision-making and the educational management of the school. This has also occurred in the school where we have carried out this study. This improves self-advocacy and empowerment (Ryan and Griffiths, 2015) and allows adults with SEN to become aware of their capabilities and what they can achieve with support from their peers and the community.

Finally, our results show that solidarity is a characteristic of the interactive learning environment studied, connected to the improvements achieved. Previous research has highlighted solidarity as one main component of interactive learning environments such as dialogic literary gatherings and interactive groups (Pulido-Rodríguez et al., 2015; Khalfaoui et al., 2020), and adult education has been identified as a context of collective action, mobilisation and solidarity leading to greater equity and inclusion (Heidemann, 2020; Smythe et al., 2021). Drawing on solidarity can contribute to approaching the right to quality inclusive education for people with disabilities at all levels and lifelong learning, as recognised in the Convention on the Rights of Persons with Disabilities (UN, 2006).

## Data Availability Statement

The raw data supporting the conclusions of this article will be made available by the authors, without undue reservation.

## Ethics Statement

The studies involving human participants were reviewed and approved by Ethics Board of the Community of Researchers on Excellence for All (CREA). Written informed consent for participation was not required for this study in accordance with the national legislation and the institutional requirements.

## Author Contributions

JD-P and EO conceptualised the research. JD-P conducted the fieldwork. MO analysed the data. JD-P and MO conducted the literature review. MO wrote a first draught of the manuscript. JD-P, EO, and AP revised and edited the manuscript. All authors contributed to the article and approved the submitted version.

## Conflict of Interest

The authors declare that the research was conducted in the absence of any commercial or financial relationships that could be construed as a potential conflict of interest.
